# The Evolution of Modern Medicine

**Published:** 1896-11

**Authors:** Joseph Eve Allen

**Affiliations:** Augusta, Ga.; Professor of Obstetrics and Pediatrics Medical Department University of Georgia; ex-President of Augusta (Ga.) Academy of Medicine, etc.


### ATLANTA
### Medical and Surgical Journal.

VOL. XIII. NOVEMBER, 1896. No. 9.

LUTHER B. GRANDY, M.D., MANAGING EDITOR.

M. B. HUTCHINS, M.D., BUSINESS MANAGER.

ALEX. W. STIRLING, M.D., C.M., M.B. (Edin.), D.P.H. (Lond.), MANAGING EDITOR PRO TEM.

COLLABORATORS:
A. W. CALHOUN, M.D., LL.D., VIRGIL O. HARDON, M.D., FLOYD W. McRAE, M.D., DUNBAR ROY, M.D., AND HENRY R. SLACK, PH.M., M.D., LAGRANGE, GA.

### ORIGINAL COMMUNICATIONS.

### THE EVOLUTION OF MODERN MEDICINE.

INTRODUCTORY ADDRESS DELIVERED AT THE OPENING OF THE SIXTY-FIFTH ANNUAL SESSION OF THE MEDICAL DEPARTMENT OF THE UNIVERSITY OF GEORGIA, OCTOBER 1, 1896.

BY JOSEPH EVE ALLEN, M.D., AUGUSTA, GA.

Professor of Obstetrics and Pediatrics Medical Department University of Georgia; ex-President of Augusta (Ga.) Academy of Medicine, etc.

Throughout all nature, in the world of mind, as well as in the world of matter, the law of evolution is universal. Its wondrous working is seen in the long succession of organic forms that culminate in the curious and complex mechanism of the human body, and its action can be traced in the progress of society from rude and uncouth barbarism to the refinement and culture of enlightened civilization. The present perfection of the arts, and the grand achievements of the sciences are all resultant from this all-pervading law of gradual development. Even in matters spiritual its operation is apparent, for a comparative study of religions shows that man in his conceptions of divinity has advanced by gradations from the grossest anthropomorphism to the most sublime ideality.

Evolution is now no longer to be regarded as the dream of cracked-brain visionaries, or a theory advocated only by advanced scientists. It is a fully established law of nature, resting upon the same truth and capable of the same demonstration as the other laws which govern the universe. I propose on this occasion to point out the influence of this law in medicine, and to study those causes and conditions which in the past have conduced to the growth of our science and the improvement of our art, and which operating in the future hold out the certain assurance of grander and more glorious attainments. The subject of my remarks to-day is

### THE EVOLUTION OF MODERN MEDICINE.

All sciences, says Compt, pass through three stages of development; first, the theological; second, the metaphysical; third, the positive stage. To this rule the medical sciences are no exception. Medicine originally was an important part of religion. Among all primitive people the office of priest and physician is always exercised by the same individual. This is true alike of the nations of antiquity and of savage and semi-civilized races now living on the globe. The connection of the priestly class with the healing art is due to the fact that when man reaches that degree of mental development that enables him to take cognizance of natural phenomena, his first impressions being ill-defined and almost always erroneous, he sees the miraculous in everything that transcends his experience or which he does not understand. Disease, which is so mysterious in causation and course is always attributed to the anger of a god, or the malice of a demon, and hence, in sickness, aid is sought of those persons who are supposed to have power over the supernatural, and to be able to propitiate the one, or checkmate the other. In the lower grades of society where all that is occult is at once charged to the caprice of beings like, though greater than man, the treatment of the sick is simply a resort to various kinds of exorcism. Modern travelers tell us that among the Indians, Tartars, Africans, and other semi-civilized races widely separated from each other, similar ideas exist as to the cause of disease, and like means are employed for its cure. Thus the medicine man, the llama, and the witch-finder endeavor to cure, by making the body of their patient intolerable to the demon that possesses it, by carrying on the most disgusting, alarming, and even painful performances. The sick person is subjected by them to dreadful sounds, fearful grimaces, and horrid odors, and is drenched with the most infernal mixtures. This idea that the efficacy of a medicine is dependent upon its repulsive qualities still survives among more enlightened people. You will find among your patients many that will value your medicine just in proportion as it is nauseating and disagreeable. A negro never thinks medicine is medicine unless it is bad to take.

From tablets, inscriptions, and other monumental remains that recent exploration has brought to light, we find that the healing of the sick by the casting out of devils was taught by the great religions of Egypt, Assyria, India, and Greece, as well as by Hebrew and Christian theology, and that the same power over disease was claimed by the priests of Osiris and Isis, of Gibil, and Æsculapius that is said to have been exercised by the priests and prophets of Jehovah.

The practice of medicine among the early Egyptians was confined to a certain order of priests who were called, in the papyri, "Masters of the Secrets of Heaven." This was a movement toward specialization, although disease was regarded as a demoniacal possession to be cured by amulets, charms, and prayers. The Prisse papryus, the oldest writing extant, is a treatise on medicine, and is a heterogeneous collection of incantations and prescriptions. The great progress that the Egyptians made in hygiene and preventive medicine under the later Pharaoh is shown by the sanitary code of the Jews, which was derived from this source. According to Herodotus Egyptian medicine in his day was entirely separated from religion, and had become highly specialized, as each department was in the hands of special practitioners, such as aurists, oculists, bone-setters, etc. On this subject, however, the Greek historian is not trustworthy.

The views of the ancient Hebrews on medical matters were not in advance of the nations around them, for in the Scriptures there is ample evidence of their belief in the supernatural origin and cure of disease. In the Old Testament the leprosy of Miriam and Uzziah, the paralysis of Jeroboam, the syphilis of Job, the dysentery of Jehoram, and the ills of many other worthies are attributed to the wrath of God, or the enmity of Satan. While in the New Testament a number of like cases are recorded, such as the sunstroke of Saul, the catalepsy of the daughter of Jairus, the epilepsy of the Gadarenes, etc. St. James positively tells us in his Epistle that the prayer of faith will save the sick. By this primitive mode of thought, this credulous grasping after the miraculous, must be interpreted the many marvelous cures that have come down to us recorded in Holy Writ.

Greece was the birthplace of scientific medicine. Here the idea of the supernatural causation of disease soon gave place to more exact knowledge.

In the temple of Æsculapius the sick were received to obtain assistance from that God. This divine help was given free of charge, but patients who recovered often, out of gratitude, made gifts to the shrine and erected votive tablets setting forth the symptoms and other particulars of their diseases. Accurate histories of cases were also kept by the priests, and it was from the study and comparison of these valuable clinical records, rather than from the celestial oracle, that guidance was obtained in the treatment of those who applied for relief. The Asclepius or Temples of Æsculapius, were devoted to both religious and medical uses, being medical schools and hospitals as well as sanctuaries.

Five hundred years before the Christian era, in the golden age of Grecian history, the struggle in medicine between science and theology began, which was continued down to the present day. The man who rose superior to the superstitions of his time and struck the first blow for liberty in science was Hippocrates, a priest of the Asclepius of Cos. Boldly he attacked and overthrew existing traditions, and laid the foundations of scientific medicine so firmly and broadly upon the impregnable principles of reason and experience that they can never be shaken and will endure forever. His works are still models of professional genius and acumen, and he will be ever venerated as the father of medicine. Hippocrates combated the theological doctrine that disease was brought about by gods or devils, and asserted that it was always due to natural causes, and was to be met only by such remedies as were dictated by observation and experiment, and not by prayers and sacrifice. Thus began that permanent separation between priest and physician that has since borne such good fruit. The memory of Hippocrates was maligned by the religious party of his day, and the charges of impiety and infidelity, that have always been heaped upon reformers of every age were made against him and his disciples. Truth, however, always prevails, and the incontrovertible logic of results has been their full and sufficient vindication.

Greece had at this time so advanced that her ancient faith was altogether inadequate to satisfy the intellectual needs of the people. The religion was fast passing away that had inspired the poet, the sculptor, and the warrior to the performance of achievements that will ever remain among the most precious possessions of mankind. A new era of thought was dawning, in which a recognition of the supremacy and sovereignty of law in the universe supplanted the chaos and confusion incident to belief to the caprices and arbitrary acts of innumerable divinities. This spectacle, often presented in the course of history, the dying out of a faith which in its day was the hope and trust of multitudes of men, is truly pregnant with suggestion and full of the most solemn import. This radical change in public opinion was inaugurated by the philosophers and physicians. The new medical philosophy contributed so much to this result, exerting, as it were, the force of a divine revelation, that the enthusiasm of Galen is almost justified when he declares that the words of Hippocrates should be reverenced as the voice of God.

The great museum of Alexandria, founded by Ptolemys Soter and Philadelphus, was the cradle of the medical sciences. Here, by patient investigation, nature, to use the quaint expression of Lord Bacon, was put to the question, and the first great discoveries in science made. In the medical school connected with this institution anatomy was recognized as the true basis of the healing art, and dissection, not only of the dead, but of the living, was practiced. The vivisection of animals and condemned criminals was here authorized by Ptolemy, who justified this apparent inhumanity by the plea that, as the culprits had already forfeited their lives, it was no injury to make them useful to the interests of society. Herophilus and Erasistratus were the great lights of the Alexandrian school, and for centuries their authority directed the course of medicine. Christianity exerted a most tremendous influence upon the evolution of medicine. For the first time in history charity became a cardinal virtue. In the early church it was the bond of unity, and was inculcated as a duty paramount to all others. The zeal for charity that possessed the first Christians manifested itself in the founding of hospitals, asylums, benevolent orders, etc., institutions utterly unknown to Pagan social economy. Then as now woman, ever responsive to the appeals of the suffering and distressed, was foremost in all philanthropic enterprises. The first public hospital was erected in Rome, during the fourth century, by Fabiola, a wealthy Roman matron, and was soon followed by hospitals in other cities, in the organization of which the Empress Helena was especially prominent. Thus a woman's sympathy, moved and guided by the world, contributed greatly to the promotion of medical science, and is one of the chief blessings of civilization.

The first hospitals and medical charities were directed by bishops and administered by deacons. This ecclesiastical control of the hospitals and the sick for a long time hindered the advance of medicine. Educated physicians were set aside because their philosophical tendencies and liberal ideas were opposed to the theology of the state, and credulity, imposture, and ignorance had full and undisputed sway. The religious dogmas of the day enforced belief in supernatural interventions, and reliance was placed in miracle, shrine, and relic cure rather than in rational medical practice. Diseases were treated entirely by saints, bones, charms, prayers, etc., remedies on a par in value with the fetishes of the African, or the totems of the Indian. The conflict between theology and scientific medicine that began in the days of Hippocrates was truly a struggle for existence during the sixteen hundred years following the conversion of Constantine. The final and lasting victory of science has just been gained in this the nineteenth century, and is a demonstration of the eternal verity of the survival of the fittest.

A dispassionate study of this conflict between science and theology is necessary to the proper understanding of the evolution of modern medicine. Theology has ever been the great stumbling block in the path of science. Religion must not, however, be confounded with, and held responsible for, the mistakes and shortcomings of theology. Theology is simply an expression of the opinions and conceptions of a group of individuals about religion. It is, therefore, ever in a state of change or evolution. Religion is constant, it is the bond that binds man to the great first cause of all creation. An impulse that permeates all nature and raises humanity nearer and nearer to the great ideal of all perfection. The faith of a people is always that to which they are mentally and physically suited, and changes with their growth in knowledge and intelligence. Men improve in their ideas of religion in direct ratio to their progress in brain development. It is entirely wrong to think that creeds are priestly inventions. The theological views of a people are the products of many underworking natural causes, the principal causes being intelligence, heredity and environment. The great agency that has refined and purified theology is science. Ever since St. Paul warned Timothy against the oppositions of science, falsely so called, there has been a great deal said about the warfare between science and religion; and with some people the term scientist has become synonymous with atheist and sceptic. Between religion and science there can never be conflict because the subject-matter of each lies in entirely different planes of thought. Science deals with phenomena altogether within the range of experience. Religion contemplates that which transcends experience, yet both science and religion meet and fully agree in the fact that the power that is manifested in the universe is forever incomprehensible to the intellect of man.

The man of science may be unable to accept current theological opinions, and yet be profoundly religious. The records of our profession are full of accounts of physicians who were regarded as atheists and infidels, and yet, whose lives of heroic devotion and self-sacrifice for their fellow-men make them the peers of any holy saint or blessed martyr that ever lived.

The adoption of Christianity by the Emperor Constantine arrested the progress of scientific medicine for nearly one thousand years. He and his immediate successors despised all learning, and repressed and stifled every form of intellectual activity; their rule plunged the world into the very depths of moral and social degradation, and ushered in the night of the Middle Ages. After his conversion Constantine, by edict, closed the Asclepius, where medicine was cultivated, prohibited the study of philosophy, destroyed the libraries and works of art and endeavored to eradicate every trace of ancient culture and civilization. The doctrine that the Bible was the sum of all knowledge, and revealed everything that was proper for man to understand, was universally accepted and effectually arrested all investigation in the field of the physical sciences and prevented advancement and discovery. A period of over sixteen centuries, a dismal blank in the history of medicine, separates Hippocrates from Paré, Herophilus from Harvey, Galen from Sydenham. It can scarcely be rightly estimated what the gain to humanity would have been had the mental activities of this period been directed to scientific research, instead of wasted in worthless theological discussion and metaphysical speculation. Many of the questions that now perplex us would have long been answered and discoveries that lie yet far in the future would have been made.

During this period the shrines of the saints were crowded by the sick and afflicted, just as the temples of Æsculapius were thronged in former times. The god had only changed his name, the worshippers were the same. It was a time of intense feeling, fierce antagonisms, and the most benighted ignorance; a time of blood and of fire, of scaffolds and stakes and instruments of torture; liberty of conscienc and freedom of speech were unknown, and the earnest seeker after truth, whether in science or religion, often fell a martyr to his enthusiasm. But for the rise of Mohammedanism all the wisdom of the ancients would have been lost forever to the world. The Arabian caliphs were ardent cultivators of learning, and especially active in the encouragement of science. This enlightened policy stands out in bold relief against the system of repression practiced by their Christian contemporaries. Under their liberal rule all the great philosophers, mathematicians, astronomers, and physicians of medieval times flourished. Thought was free and unshackled, and a man was at liberty to learn and believe whatsoever he chose.

Wherever Saracen conquest reached the physicians were the leaders of thought and the medical schools were the centers of intellectual enlightenment.

From the study of medicine by the Arabians many other sciences, such as chemistry, botany, and metallurgy, were developed. The Arabians acquired much of their knowledge of medicine from the Nestorians and the Jews. These outcasts of Christendom found under the protection of the Crescent the security and encouragement so necessary to scientific pursuits. They gave especial attention to the investigation of the structure and diseases of the human body, and were noted for their skill and learning as physicians. Jewish and Nestorian physicians founded the celebrated medical school of Djondesabour, the first college to institute the system of academic degree which we still have to-day. They also founded universities at Damascus and Bagdad, and in Moorish Spain. Thus they were the medical teachers of the Moslems and afterwards of Christian Europe. To the persecution of these heretics by Christian fanatics, therefore, science owes the rich treasures of Arabian learning.

Although the Arabians were generally liberal in their views, and favorable to the free expression of thought and opinion, still there were bigots and religious fanatics among them as well as among other nations, as is evidenced by the treatment accorded to Averroes, the greatest philosopher and physician of the eleventh century, who was for years subjected to great persecution, and even, at one time, was forced to sit upon the steps of a mosque in Cordova, to be spit upon by the faithful because of his scientific opinions.

Jewish physicians were always prominent in the cause of medical education. They founded, in 1140, the first medical school in Europe, the School of Salerno, and subsequently the Medical College of Montpellier. During the middle ages they were much sought after because of their great ability, and although they were under the ban of the church, and cursed as infidels and unbelievers, still they were often the trusted friends and attendants of princes, prelates, and even popes. To their everlasting credit be it said that the influence thus acquired was always exerted for the betterment of mankind. The eminence of Jewish physicians was only attained after a hard struggle with ecclesiastical authority within the bounds of their own faith. The Levites claimed the divine right to heal the sick by prayers and expiatory sacrifice, and only surrendered this prerogative after the most obstinate resistance. The beginning of the separation of the priest from the physician in Judaism is first noted in the book of Ecclesiasticus, which, though apocryphal, reflects correctly the ideas of the age in which it was written.

The evolution of scientific medicine has been one continuous struggle with dogmatic theology. For two thousand years no new principal in medical philosophy has even been proposed, or advance in practice advocated, without meeting with the most determined opposition from persons speaking in the sacred name of religion.

Theologians have always regarded the progress of science with the greatest antagonism; this is because of the many assaults that scientists have made upon their interpretations of nature, and the fear that perhaps sooner or later all that they hold as necessary to salvation may be swept away. Criticism and examination only more firmly established the truths of science, while many of the most cherished doctrines of theology have been disproven and shown to be erroneous by the advance of knowledge. From age to age this conflict has gone on. In every contest science has been the victor and theology has been forced to give up one after another of its positions, but has defended those that remain with undiminished tenacity, and, defeated at last, has always been ready with a plausible compromise.

The progress of science, however, no matter how dangerous to religion some of its stages may have seemed for the time to be, has invariably resulted in the highest good both of religion and science. The truth of this is fully illustrated in the history of medicine by the contests over miracle cure, demoniacal possession, the practical study of anatomy, the use of anesthetics, the treatment of the insane, public sanitation, etc.

### MIRACLE CURE VERSUS MEDICINE.

Under all religions persons who have been great benefactors to their race have always been credited with miraculous powers of healing; this is true of Brahmins, Buddhists and Mohammedans, as well as Christian saints. Belief in these cures were formerly unquestioned and sustained by the most ridiculous and incompetent testimony. Every church and town in Christendom had its miracle-working relics, holy well, or grotto, to which the sick flocked in multitudes for cure. Over ninety per cent. of the afflicted prayed at these shrines without avail, yet faith was not disturbed. Those cures that were effected were clearly the work of the imagination, as were those worked in more recent times by John Wesley, Schlatter, Ivan, and others. This fetishism, which for ages obstructed the advance of rational medicine, was based on, and sustained by, scriptural authority, for was not Naaman cured by a bath in the river Jordan? Were not those healed who touched the bones of Elisha? Did not St. Peter, by faith, raise the cripple at the Temple gate, and why could not other blessed waters and the bones of other holy persons be just as efficacious? Relic and shrine cure was insisted on as a religious duty, and the biblical case of King Asa, who died because he trusted in the physicians and not in the Lord, was often quoted with great unction in its support. The delusion of miracle cure was gradually dispelled by the rise of secular medical schools, such as those of Salerno, Montpellier, Bologna, etc., and the introduction of Arabian learning through the Wars of the Crusades.

The passage from miracle to medicine was very slowly effected, for these superstitions were deeply rooted in the minds of men, and only given up with the greatest reluctance. The discoveries that brought about this result were, first, those in biology relating to the mechanism of the human body; second, those in pyschology showing the connection of the mind with the body, such as the influence of the imagination, the effect of hypnotism, suggestion, hysteria, etc.; third, those in pattology and bacteriology bearing upon the nature and causes of disease.

### THE GENESIS OF SURGERY.

Surgery, which is to-day one of the foremost of the medical sciences, in the early stages of the evolutionary process was altogether distinct from the practice of medicine. In the beginning the doctor was an entirely different personage from the surgeon. The first was supposed to treat ills that were of supernatural origin, while the other cured those afflictions, the causation of which was obvious. The doctor's practice was confined to internal diseases which were attributed to divine displeasure, demoniacal possession, etc. The surgeon treated wounds and other injuries received in battle, the chase, or through accident. In the middle ages when medicine was altogether under the control of the church, ecclesiastics did not perform surgical operations, but simply superintended those done by assistants who were laymen, because the church law which prohibits the clergy from shedding blood prevented them from using the operating-knife. In the fifteenth century the practice of surgery was a despised and disreputable calling, left altogether in the hands of the barbers, and was so neglected that many dignitaries in church and State perished for the want of the simplest surgical operation. To the labors of Paré, Vesalius, and Harvey is due the impulse that through succeeding ages has brought about the present high status of the science and art of surgery.

### DEMONIACAL POSSESSION AND THE TREATMENT OF THE INSANE.

To the teachings of St. Paul is due the first infusion of pagan ideas into Christianity. To him the gods of the heathen were real beings, for in his Epistle to the Corinthians he says that "the things which the gentiles sacrifice, they sacrifice to the devil and not to God." Based upon this the belief became general that diseases, especially those of the brain, were a kind of demoniacal possession. This belief in the devil and his workings in the physical organism of man is supported by overwhelming scriptural evidence and by the unanimous authority of the early fathers of the church

The Reformation did not altar in the least the views of the people on this subject, for since the day of Luther who said that "Satan produces all the ills that afflict mankind," down to the humbuggery of faith-cure, Christian science, etc., of our times, Protestantism has always held and encouraged the idea of the supernatural origin of disease. By the dictim of theology lunatics were for nearly two thousand years treated by exorcisms and punishments. They were chained in foul, dark dungeons, flogged, starved, and prayed over; for it was thought highly meritorious to inflict pain on these poor wretches, because by so doing the devil that possessed them was tormented. Although science has, ever since the days of Hippocrates, asserted that madness was a physical ailment, it was not until the end of the last century that this inhumanity was arrested. In 1792 Piner struck off the chains from the lunatics in the hospitals of Paris and inaugurated a new era in the treatment of insanity. Science has cast Satan out of the lunatic asylums and will soon run him off the face of the earth.

THE DECLINE AND FALL OF CHURCH RULE IN MEDICINE.

In Christendom the emancipation of medicine from ecclesiastical control did not take place until a late period. At first the treatment of the sick was altogether in the hands of the clergy, some of whom, notably the Benedictine monks, became quite celebrated for their skill in medicine. As late as 1452 physicians in France were not permitted to marry, because they were considered as belonging to the clergy, and in Henry VIII.'s reign no person could practice physic in England without examination and license from the Bishop of London, or the Dean of Paul's, duly assisted by the faculty. The Archbishop of Canterbury had the latent right to grant medical diplomas up to the opening years of the present century. The separation of the soul-curer from the body-curer, the priest from the physician, was brought about at last by the general enlightenment of society following the invention of the art of printing. Before this time it is true that the practice of medicine by ecclesiastics had fallen into discredit, and various church councils had condemned it as irreligious and highly atheistical; declaring it to be the greatest impiety to seek relief in other than those supernatural means that religion had made so abundant and easily accessible. When the craze for relic cure was at its height, invalids went the rounds from one shrine to another, just as they go to-day from one specialist to another, and from one sanitarium to another. Certain relics for a time were more fashionable than others, and some would rise in popularity for a period and then relapse into innocuous desuetude. Some relics cured one disease and were not good for another. Thus St. Gall cured tumors, St. Christopher throat diseases, and St. Remy fevers, etc. The growth of medical knowledge after a time enabled physicians to cure diseases that relics could not reach. The success was at once attributed to the assistance of the devil. And even as late as the seventeenth century, when the Town Council of Hall in Würt-temberg gave privileges to a Jewish physician on account of his admirable skill and ability, the clergy of that city joined in a protest, stating that "it were better to die with Christ than be cured by a Jew doctor aided by the devil." As people became better educated the true value of science was more and more appreciated, until, at last, church rule in medicine has passed away forever.

### THE BATTLE FOR DISSECTION.

The study of anatomy by dissection was in Europe for centuries proscribed by Christian ecclesiastical authority. This opposition to dissection was probably inherited from earlier times, because the mutilation of the dead was held as desecration and sacrilege by all ancient religions. Even the old Egyptians who considered the embalming of the body necessary to its resurrection, still despised the embalmer and his art. This prejudice against dissection was, however, in a great measure overcome by the influence of the Alexandrian school, and at the time of the conversion of the Roman Empire to Christianity anatomy was extensively cultivated as an important branch of medicine. The doctrine that the human body was made in the likeness of God and was the temple of the Holy Ghost caused the early Christians to condemn dissection, and when in the eighth century the formula of Marcellus, now known as the Apostle's Creed, was adopted as orthodox belief, supplanting and contradicting St. Paul's view of the resurrection, the cause of anatomy received a stunning blow from which it did not recover for hundreds of years. It was argued that injury to the body might in some way prevent its rising on the day of judgment, and that hence its dissection was a crime deserving of punishment here and hereafter.

The struggle for anatomy began in the thirteenth century when the heretic Emperor Frederick of Germany granted his physicians the privilege of dissecting the human subject. Before this time the universities of Bologna, Salamanca, Paris, and possibly some others, taught anatomy by using apes and monkeys as dissecting material, a very good substitute seeing how near these animals are related to the *genus homo*. In 1391 the University of Lerida was allowed to dissect one dead criminal every three years. Paracelsus, in the early part of the sixteenth century, did much to prepare the way for the proper appreciation of anatomy. The discovery by Servetus of the lesser or pulmonary circulation was, however, the first great advance. This man perished a victim of religious fanaticism, for he, together with the book containing the proof of his great discovery, was burned at Geneva in 1553 through the machinations of that greatest Protestant reformer, John Calvin. It was Andreas Vesalius, in the sixteenth century, who won the battle for science and founded modern anatomy. Single-handed and alone he fought the good fight. Despising ridicule and slander and opposition, ostracised by his professional brethren and excommunicated by his church, he labored on, regarding not the peril nor the sacrifice. Carried away by enthusiasm in his science, he renounced all the pleasures of life and to obtain subjects for study lived among the gibbets and the tombs. With dauntless heroism he defied alike the fires of the inquisition and the contagion of the plague, until finally borne down by persecution he fell a martyr to science and humanity, and left a name and an example that will be forever a stimulus and an inspiration to laborers in the cause of truth. From the time of Vesalius dissection has been everywhere recognized as a necessary part of a medical education, but only within the last twenty-five years have the senseless laws been annulled that prevented the getting of anatomical material.

The medieval anatomists wasted a great deal of time looking, without success, for a hypothetical structure known as the resurrection bone, which was supposed to be a bone imperishable and imponderable, that was to form the nucleus of the new body on judgment day. During later times the special object of anatomical search was for the seat of the soul. The brain and heart were the fields for the particular inquiry. For a long time the soul was supposed to reside in the pineal gland; afterwards it was asserted that its habitat was the hippocampus minor, and this was the generally received belief among theologians until Huxley disproved it, by showing that this structure exists in the anthropoid apes as well as in man. The seat of the soul is still an anatomical *terra incognita*, and is likely to remain so.

### FETISHISM VERSUS SANITATION.

The security and protection afforded against infectious and contagious diseases is one of the great triumphs of modern medicine. In ancient times, and even down to very recent periods, nations were often visited by fearful epidemics that wrought havoc and devastation and swept off thousands of victims. By both pagans and Christians these pestilences were attributed to the wrath of God, against which vain was the help of man. They were regarded as scourges only to be averted by prayer, fasting, and offerings. On the appearance of an epidemic repentance and atonement for sin were preached, and no effort was made to arrest the spread of the disease by the adoption and enforcement of the most obviously necessary sanitary measures. The belief in the supernatural cause of epidemics checked and paralyzed scientific effort and resulted in terrible mortality. The loss of life was enormous, and was entirely due to the neglect of proper hygienic precautions. In Europe during this period the masses lived crowded together in filth and wretchedness, and in the habitation of the rich no attention was paid to sanitation or cleanliness. In the cities garbage was allowed to accumulate in the streets and sewage and drainage was unknown. The earthen floors of the dwellings were often one mass of decomposing organic matter, affording an inviting culture-field for every variety of pathogenic germ, and it is not to be wondered at that with such favorable surroundings these germs should have multiplied and flourished, notwithstanding the prayers and supplications of the faithful. The reason for this state of things was because filthiness was considered by the early Christians as a repudiation of heathenism, for the old Greeks and Romans having learned, at great cost, the importance of cleanliness to public health, enforced it as a religious duty. During the middle ages the idea became prevalent that abuse of the body secured the salvation of the soul. This encouraged the tendency to ignorance and sloth that is inherent in human nature and led to the production of dirty personal habits that greatly facilitated the spread of epidemics.

Sometimes other means besides fasting and prayers were employed to arrest epidemics. In 1522 in Rome, the center of the Christian world, thirty-two years after the discovery of America, an ox was sacrificed in the Colosseum, to the heathen gods in the hope that by propitiating them the plague that was then raging might be stayed. During the epidemics known as the black death the Jews, owing to the observance of the sanitary laws laid down by Moses, frequently escaped the disease. This immunity was at once attributed to the especial protection of the devil and the effort was made to stop the epidemic by torturing and murdering that unoffending people. Plagues were often supposed to be due to witchcraft, and thousands of innocent men, women, and children were executed as the witches through whose sorcery these diseases originated. This was a very favorite method of stopping epidemics, justified by the scriptural injunction, "Thou shalt not suffer a witch to live."

But for the theological bias the real cause of the spread of epidemics would have soon been recognized and understood, for often it was very near to being discovered. Thus in ancient times contaminated water supply was frequently charged with being the source of these diseases, but this was soon lost sight of, and the idea of supernatural causation was generally accepted. At last men gradually awakened to the truth of John Wesley's maxim, that "Cleanliness is akin to Godliness," and realized that the sin for which infectious and contagious diseases is the punishment is the unpardonable sin of filthiness.

In our time knowledge has so advanced on these subjects that the carriers of contagion are now known by all to be material in character, and we hear nothing of visitations, scourges, inscrutable providences, etc., in this connection. The labors of Jenner in the last century, and of Charles Chadwick in this, have contributed greatly to the education of the people, which has resulted in the control of epidemic diseases.

As an illustration of present opinion on this subject two recent incidents may be cited. Thus, when Lord Palmerston, who was then Premier of Great Britain, was requested by the Scotch clergy to appoint a fast day to ward off the cholera, he refused, and told them to go home and clean their streets. And the present emperor of Germany, in the face of a similar emergency, forbade prayer-meetings because they diverted people from the employment of proper sanitary measures.

Statistics conclusively show that fifty years of scientific sanitation has done more to prevent disease and death than fifteen hundred years of prayer and religious observance. If, therefore, science has accomplished so much, may we not reasonably hope that through its further progress, not only plagues and pestilences, but such diseases as typhoid fever, phthisis, diphtheria, pneumonia, etc., will also soon be banished from the earth.

We are now living in an epochal period in the history of medicine. A period marked by wonderful improvement in diagnostic precision, by a great advance from empiricism to positive therapeutics, by astonishing results in surgery through asepsis and antisepsis, by fruitful and far-reaching discoveries in bacteriology and pathology, by rapid progress in sanitation, hygiene, and preventive medicine, and by increased knowledge in every branch of the healing art.

It is not these achievements, however, laden though they be with manifold blessings to mankind, that will distinguish the closing years of the nineteenth century. The achievement that rises far above them all, and which in the future offers the possibility and the assurance of great and permanent advancement is this, that medicine has, at last, in every department thrown off the yoke of mystery and superstition, and faces the new era forever free and untrammeled by the chains of dogma and tradition.

